# Hypoxia-inducible factor-2 alpha up-regulates CD70 under hypoxia and enhances anchorage-independent growth and aggressiveness in cancer cells

**DOI:** 10.18632/oncotarget.24919

**Published:** 2018-04-10

**Authors:** Shojiro Kitajima, Kian Leong Lee, Masaki Fujioka, Wendi Sun, Jia You, Grace Sushin Chia, Hideki Wanibuchi, Shuhei Tomita, Marito Araki, Hiroyuki Kato, Lorenz Poellinger

**Affiliations:** ^1^ Cancer Science Institute of Singapore, National University of Singapore, Singapore, Singapore; ^2^ Department of Pharmacology, Graduate School of Medicine, Osaka City University, Osaka, Japan; ^3^ Cancer and Stem Cell Biology Program, Duke-NUS Medical School, Singapore, Singapore; ^4^ Department of Molecular Pathology, Graduate School of Medicine, Osaka City University, Osaka, Japan; ^5^ School of Biological Sciences, Nanyang Technological University, Singapore, Singapore; ^6^ Department of Transfusion Medicine and Stem Cell Regulation, Juntendo University Graduate School of Medicine, Tokyo, Japan; ^7^ Department of Cell and Molecular Biology, Karolinska Institutet, Stockholm, Sweden

**Keywords:** hypoxia, HIF-2α, CD70, epigenetics, DNMT1

## Abstract

Hypoxia-inducible factors (HIFs) facilitate cellular adaptation to environmental stress such as low oxygen conditions (hypoxia) and consequently promote tumor growth. While HIF-1α functions in cancer progression have been increasingly recognized, the contribution of HIF-2α remains widely unclear despite accumulating reports showing its overexpression in cancer cells. Here, we report that HIF-2α up-regulates the expression of CD70, a cancer-related surface antigen that improves anchorage-independent growth in cancer cells and is associated with poor clinical prognosis, which can be induced via epigenetic modifications mediated by DNMT1. The ablation of CD70 by RNAi led to decreased colony forming efficiency in soft agar. Most strikingly, we identified the emergence of CD70-expressing cells derived from CD70-negative cell lines upon prolonged hypoxia exposure or DNMT1 inhibition, both of which significantly reduced CpG-nucleotide methylations within CD70 promoter region. Interestingly, DNMT1 expression was decreased under hypoxia, which was rescued by HIF-2α knockdown. In addition, the expression of CD70 and colony forming efficiency in soft agar were decreased by knockdown of HIF-2α. These findings indicate that CD70 expression and an aggressive phenotype of cancer cells is driven under hypoxic conditions and mediated by HIF-2α functions and epigenetic modifications. This provides additional insights into the role of HIF-2α in coordinated regulation of stem-like functions and epigenetics that are important for cancer progression and may present additional targets for the development of novel combinatorial therapeutics.

## INTRODUCTION

The core regulatory mechanisms for oxygen sensing and adaptation to hypoxia have widely been identified in the past decades [1–4], whereby the hypoxia-inducible factors HIF-1α and HIF-2α that are oxygen-sensitive subunits of transcriptional complexes predominantly mediate the adaptive responses. Although the HIFs are activated in a similar manner and their transcriptional targets are partially overlapping, HIF-1α and HIF-2α have a striking divergence in function [5]. Recent reports have implicated HIF-2α function in tumor progression [6, 7] and especially in the regulation of cancer stem cells [8, 9]. The role of HIF-2α in cancer, however, widely remains an area of intense interest and has yet to be fully elucidated.

As tumors expand, genetic and/or epigenetic alterations generate cellular heterogeneity [10–12], which gives rise to subpopulations that develop growth advantage or chemoresistance [13]. Notably, tumor hypoxia and HIFs have been engaged in epigenetic regulations via remodeling of DNA methylation by DNMTs [14, 15] and up-regulation of the Jumonji C (JmjC)-domain containing histone demethylases [16–18]. Therefore, identifying epigenetic regulations under hypoxia that generate population diversity and elicit cellular aggressiveness may expand our understanding of cancer cell heterogeneity leading to additional avenues for treatment.

CD70 (*TNFSF7*) was identified as a member of the tumor necrosis factor (TNF) superfamily and associated with the regulation of lymphocytes via its counterpart protein CD27 [19, 20]. The importance of CD70 in lymphocyte and leukemia cell activation has been investigated in earlier studies [21, 22], while the expression and emerging roles (e.g. immune escape) of CD70 have also been reported in various solid tumors [23–28]. Due to the prominent role of CD70 in cancer, the applications for CD70 targeted therapy have also been developed [29–31]. Although some studies suggested that CD70 is regulated by epigenetic mechanisms [32, 33], how it is governed in response to the alterations in the tumor microenvironment has yet to be determined. Here, we report an involvement of the microenvironmental parameter hypoxia and the hypoxia-regulated HIF-2α transcription factor in the epigenetic regulation of CD70 expression, which may contribute to enhanced anchorage-independent growth of cancer cells.

## RESULTS

### CD70 expression is indicative of poor prognosis in diverse cancers

**Figure 1:**
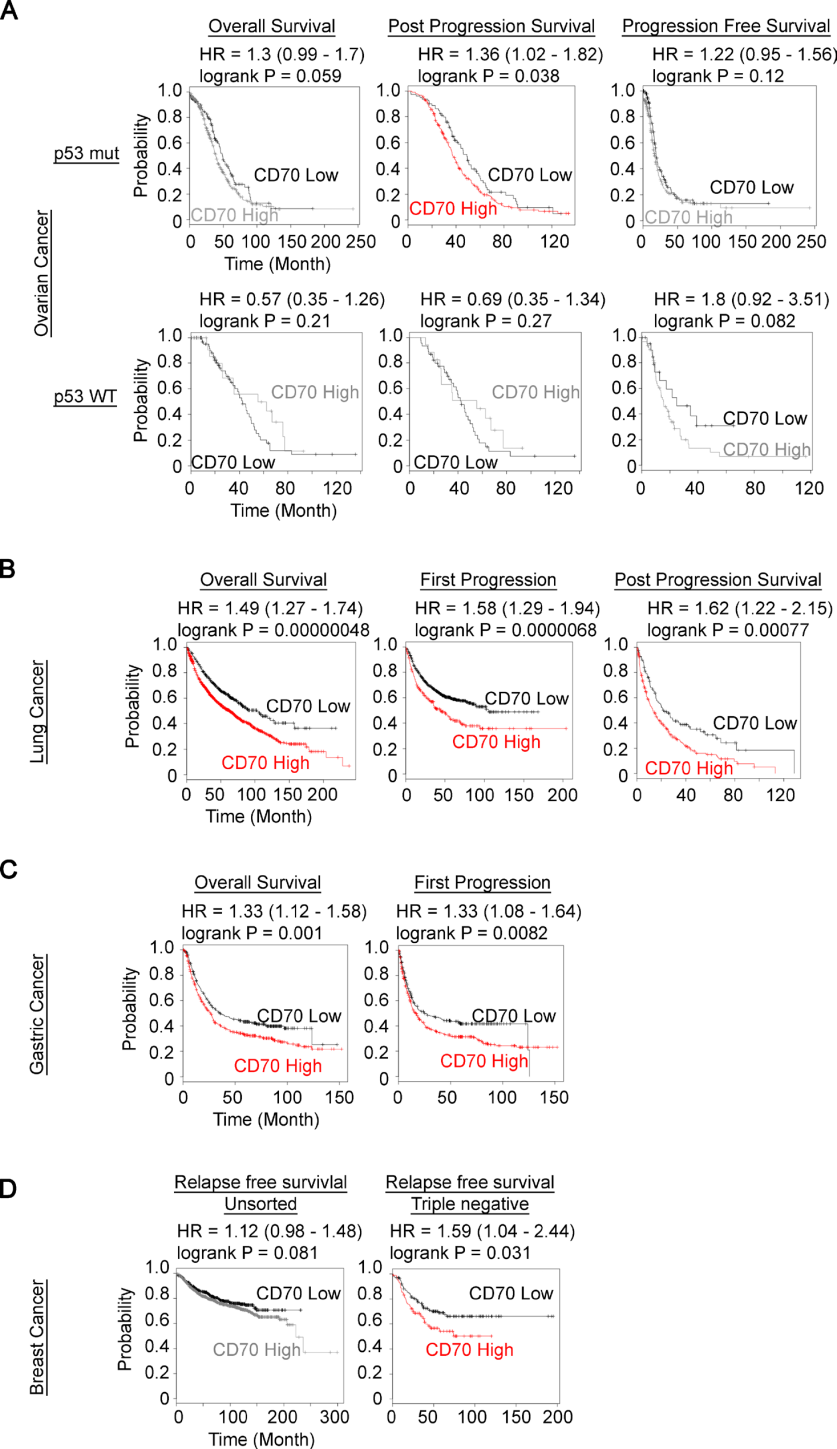
Prognostic value of CD70 expression in human cancer patient (**A**–**D)** Kaplan–Meier plots of CD70 expression in tumors with patient survival as indicated were generated using the KM plotter (kmplot.com). (A) ovarian cancer with wild-type (WT) or mutant (mut) p53 status. (B) lung cancer. (C) gastric cancer. (D) unsorted breast cancer (left) and triple negative (right) breast cancer. The colored plots show statistically significant differences between the groups.

Although ectopic expressions of CD70 in solid cancer types and hematopoietic malignancies have been established and applications of neutralizing antibody for CD70-targeted therapeutics have been tested in various mouse tumor models [29, 30, 34, 35], limited studies have reported the association between CD70 expression and the survival rate of cancer patients [36]. Hence, we evaluated the prognostic values of CD70 expression on clinical outcome through the use of primary patient gene expression databases [37, 38]. We observed correlations between high *CD70* mRNA expression and poorer prognosis in ovarian, lung, gastric and breast cancer patient but not in glioma cases (Figure 1A–1D and Supplementary Figure 1A). We next determined CD70 protein expression and how this correlates with the efficiency of anchorage-independent growth in human cancer cell lines. Interestingly, a marked trend of a higher colony number in soft agar in CD70-positive (CD70^+^) cells compared to CD70-negative (CD70^–^) was identified in 9 ovarian, 5 lung, 2 kidney and 2 brain cancer cell lines in soft agar assays (Table 1). These findings collectively suggest that CD70 may serve as a potential marker for clinical and cellular aggressiveness of diverse cancers.

**Table 1:** Expression of CD70 and colony forming efficiency in soft agar of cancer cell lines

	Organ	CD70	Colony		Organ	CD70	Colony
PEO1	Ovary	+	+	A549	Lung	+	+
TOV-21G	Ovary	+	++	H1299	Lung	+	+
TOV-112D	Ovary	+	++	H1975	Lung	+	+
SK-OV-3	Ovary	+	++	HCC2935	Lung	−	−
CaOV-2	Ovary	+	++	PC-14	Lung	−	−
HEYA8	Ovary	+	+	LN229	Brain	+	++
OVTOKO	Ovary	−	−	A172	Brain	−	−
OVISE	Ovary	−	−	786-O	Kidney	+	−
PA-1	Ovary	−	−	A498	Kidney	+	++

Colony number; –< 20 +20 ≤, < 60 ++ ≥ 60.

### CD70 enhances anchorage-independent growth in ovarian and lung cancer cells

**Figure 2:**
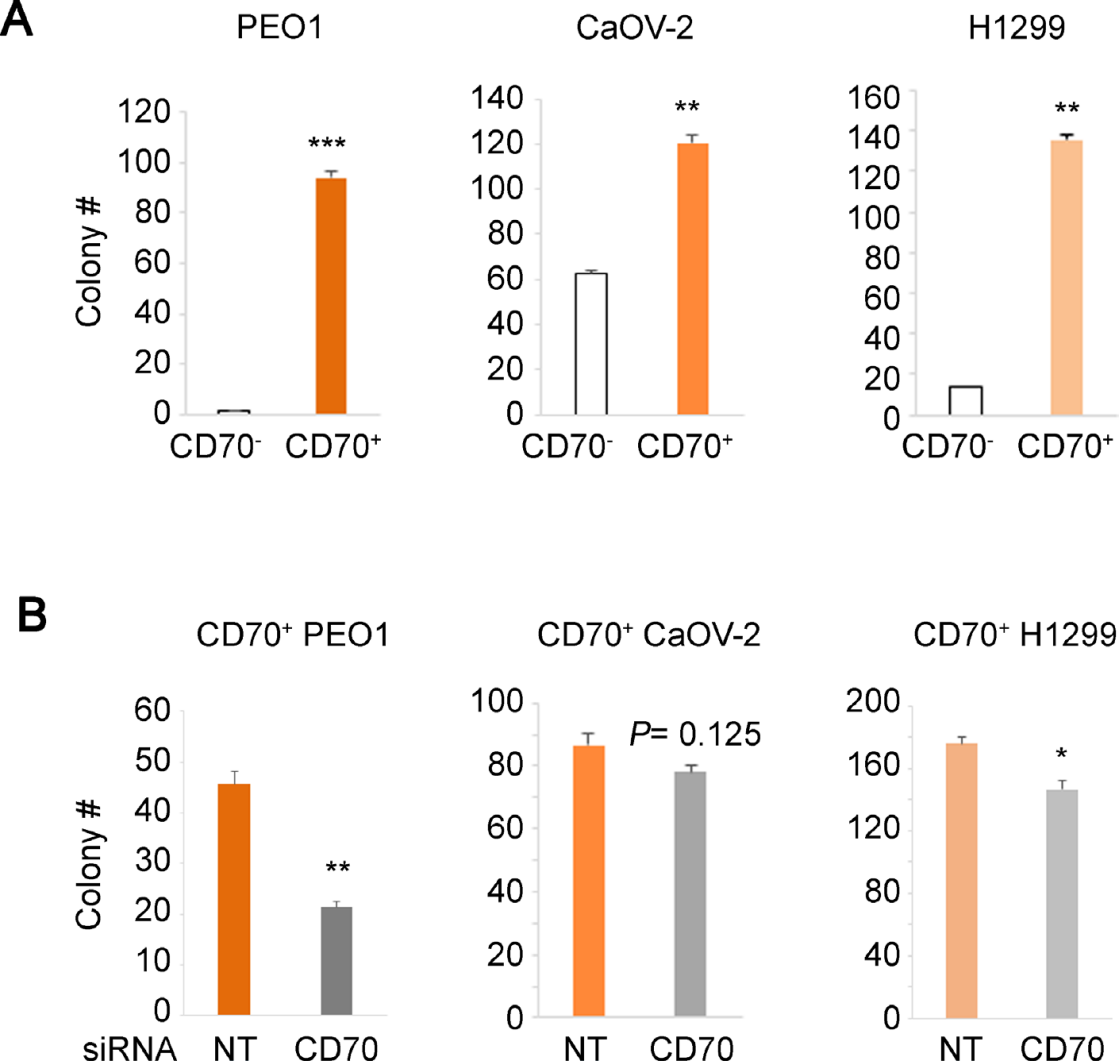
Correlation between CD70 expression and colony forming efficiency in soft agar (**A**) colony numbers of FACS-sorted and established CD70^+^ or CD70^–^ cells from each cancer cell line as indicated were obtained using soft agar assays. (**B**) the effect of CD70 knockdown on anchorage-independent growth. Each cell line was transfected with siRNA against CD70 or the non-targeting (NT) control and grown in soft agar. Error bars indicate s.e.m. ^*^P < 0.05; ^**^P < 0.01; ^***^P < 0.001 (Student’s *t*-test).

Given the statistical significance of CD70 in poor patient survival described above, we further examined whether CD70 expression promotes cancer cell growth. Protein expression profiles and cellular properties vary intrinsically between cell types of different origins. Hence, to control for this variation, we sorted CD70^+^ and CD70^–^ cells from the ovarian cancer cell lines PEO1, CaOV-2 and lung H1299 and compared their growth properties in soft agar to determine the effects of presence or absence of CD70. Interestingly, the CD70^+^ cells in all 3 cell lines exhibited significantly higher colony forming potential compared to CD70^–^ cells (Figure 2A). To validate the importance of CD70 in increased anchorage-independent growth of cancer cells, we next carried out siRNA knockdown of CD70 expression. The ablation of CD70 by siRNA resulted in reduced colony numbers in PEO1 and H1299 cells while the effect was not statistically significant in CaOV-2 (Figure 2B and Supplementary Figure 1B). Consistently, the attenuated anchorage-independent cell growth was confirmed by shRNA-CD70 in CD70^+^ PEO1 cells (Supplementary Figure 1C). These indicated the dependence of cancer cell growth on CD70-associated pathways. CD70^−^ CaOV-2 cells were able to form a large number of colonies although the CD70^+^ cells showed significantly higher colony forming efficiency. This suggests that CD70 dependency in CaOV-2 cells may be lower compared to the other 2 cell lines. Anchorage-independent growth is a complex multilateral cellular activity that involves suppression of anoikis and many other pathways. Our data collectively suggest that CD70 at least in part contributes to anchorage-independent growth and may account for its role in oncogenic aggressiveness.

### CD70 is induced both by chronic hypoxia and DNMT1 inhibition via alteration of DNA methylation status

**Figure 3:**
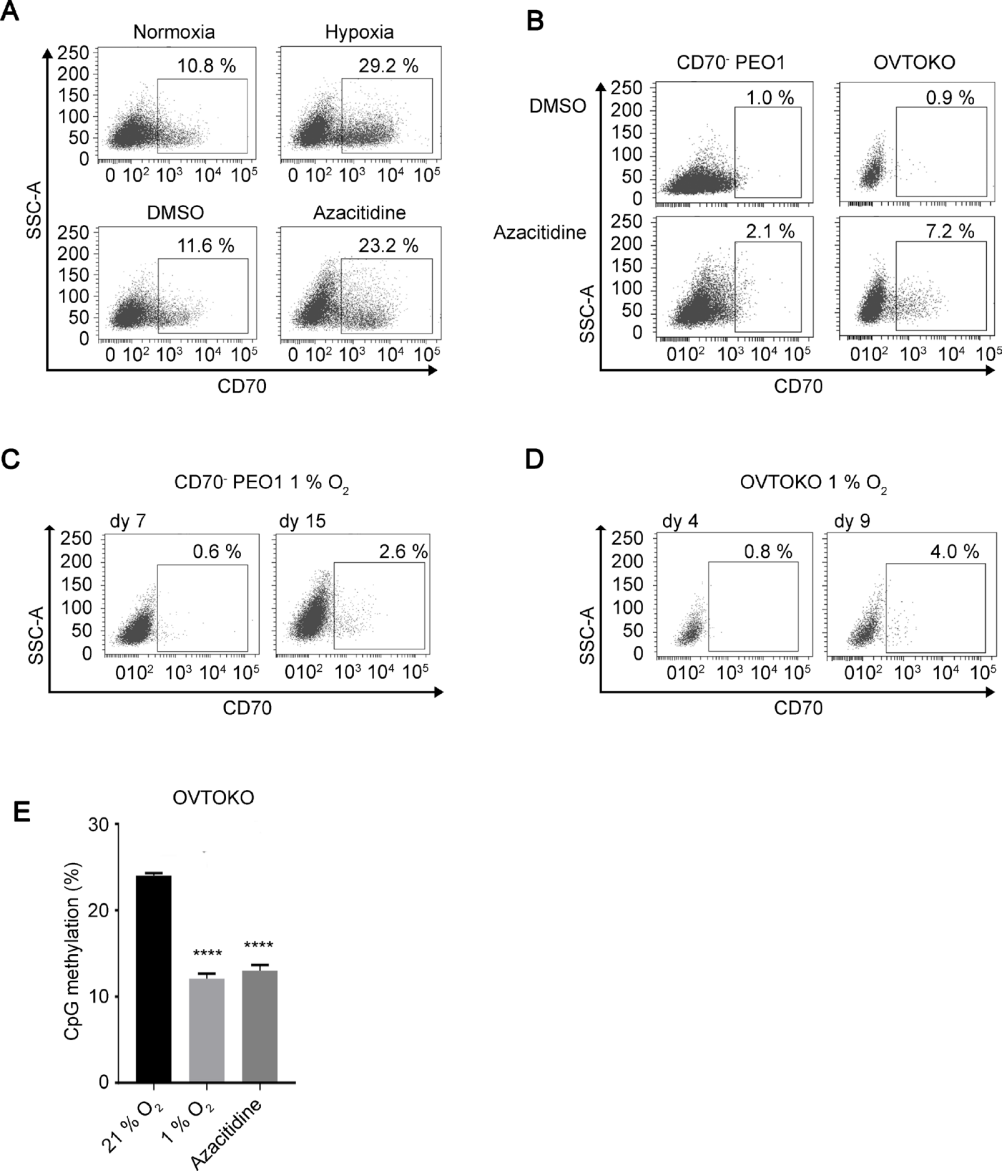
CD70 is regulated by DNMT1 and oxygen tension (**A**) the effect of oxygen levels (upper panels) or DNMT1 inhibition (azacitidine, lower) on CD70 expression in PEO1 cells was analyzed by flow cytometry. Each treatment was performed for 5 days. (**B**) induction of CD70 positive cells from sorted CD70^–^ PEO1 (left panels) and OVTOKO (right) cells by an 8-day azacitidine treatment. (**C**, **D**) detection of CD70^+^ cells derived from CD70^–^ PEO1 (C) and OVTOKO (D) cells after long-term hypoxia treatment (1% O_2_). (**E**) Bar chart shows the levels of methylated CpG dinucleotides within CD70 promoter region. Error bars indicate s.e.m. ^****^P < 0.0001 (Student’s *t*-test).

Increased colony forming ability in soft agar is an important property of cancer stem cells [39, 40], and has also been reported to be driven in hypoxic niches [41]. To address this, we next examined if CD70 expression is regulated by hypoxia. Interestingly, the CD70^+^ cell population in PEO1 cells increased by about 18.4% under 1% O_2_ conditions (hypoxia) for 5 days compared to 21% O_2_ (normoxia, Figure 3A upper panels). Our previous study and many others have shown that a great number of genes are epigenetically regulated under hypoxia [14, 42, 43]. Indeed, we found increased numbers of CD70^+^ cells during DNMT1 inhibition by a 5-day 5-azacitidine treatment that has the potential to revert epigenetic silencing mediated by DNA methylation (Figure 3A lower panels). These data suggest that CD70 expression may be epigenetically derepressed by hypoxia. It is possible that the elevated CD70^+^ cell ratio could have been caused by population changes in response to cellular stresses as PEO1 cells are a mixture of CD70^+^/CD70^–^ cells. Therefore, we next employed the sorted and established CD70^–^ PEO1 cells and OVTOKO cells that were also CD70^–^. Both cell lines demonstrated the induction of CD70^+^ cells from the pure CD70^–^ populations during an 8-day DNMT1 inhibition (Figure 3B). In particular, long-term hypoxia treatment also resulted in the appearance of CD70^+^ cells from CD70^–^ PEO1 and OVTOKO cells (Figure 3C and 3D). These imply that a change in DNA methylation status may be responsible for CD70 induction. Thus, we next tested the DNA methylation status of CD70 in the presence or absence of long-term hypoxia or DNMT1 inhibition. Strikingly, either 8 days-incubation under 1% O_2_ or 5-azacitidine treatment significantly decreased the promoter methylation of CD70 in OVTOKO cells (Figure 3E). These findings collectively suggest an involvement of DNMT1 in the regulation of CD70 under hypoxia. Taken together, these studies indicate that chronic hypoxic conditions allow CD70^+^ cells to emerge from CD70^–^ populations and this is likely mediated by epigenetic modifications in cancer cells.

### Hypoxia-inducible factor-2α plays an important role in the regulation of CD70 under hypoxia

**Figure 4:**
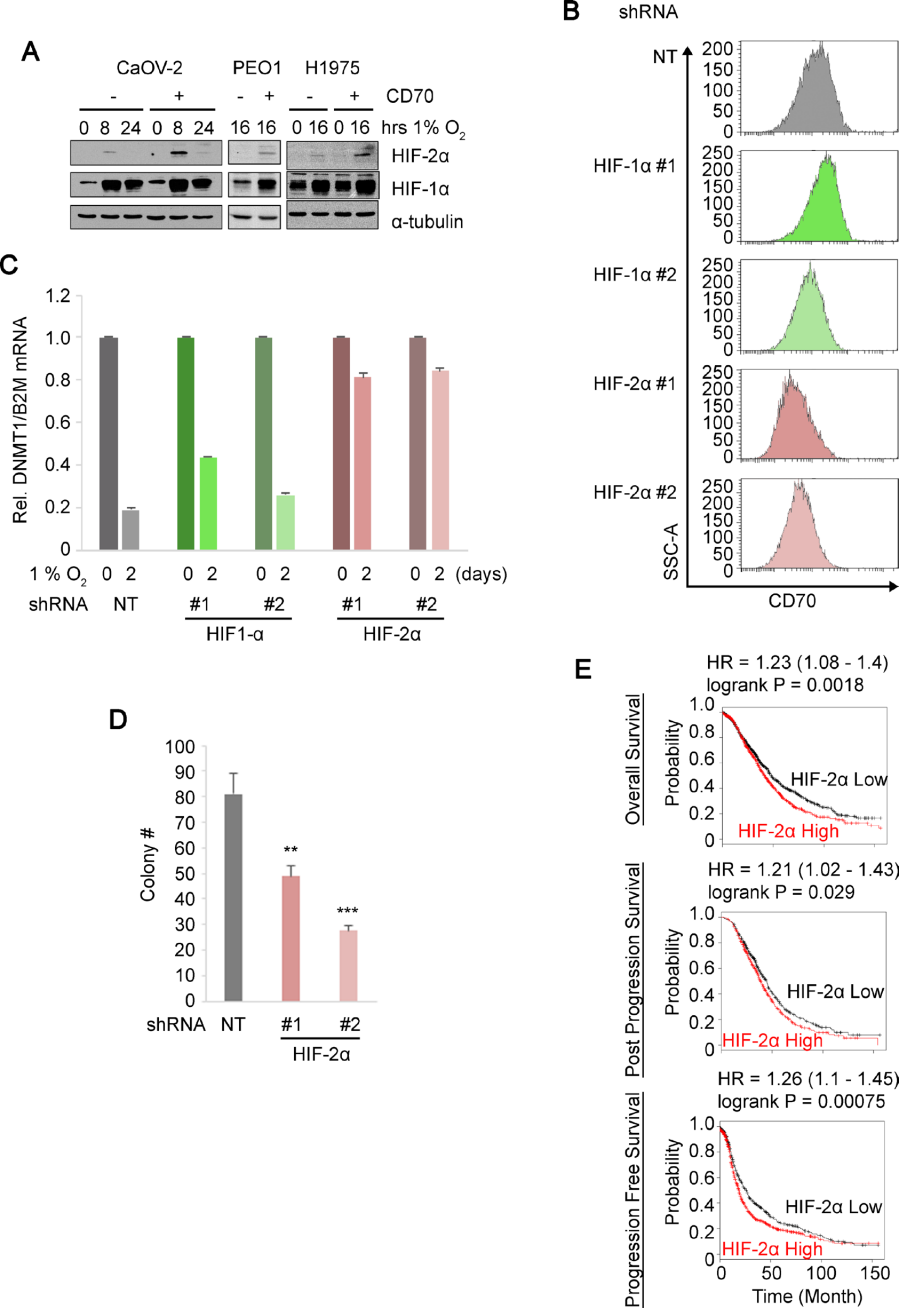
HIF-2α regulates CD70 expression and anchorage-independent growth (**A**) immunoblots comparing HIF-2α and HIF-1α expressions in the sorted CD70^+^/CD70^–^ ovarian/lung cancer cell lines under hypoxia at the time points indicated with α-tubulin as loading control. (**B**) histograms show CD70 expressions in non-targeting (NT) control, HIF-1α or HIF-2α knockdown of CD70^+^ PEO1 cells cultured under hypoxic conditions for 5 days. (**C**) DNMT1 mRNA expression levels normalized to housekeeping reference B2M in HIF-1α/HIF-2α knocked-down CD70^+^ PEO1 cells with NT control. (**D**) bar chart indicates colony numbers in soft agar by two independent shRNAs against HIF-2α or NT control in CD70^+^ PEO1 cells. (**E**) Kaplan–Meier survival curves comparing high and low HIF-2α expression in ovarian cancer cases. Error bars indicate s.e.m. ^**^P < 0.01; ^***^P < 0.001 (Student’s *t*-test).

We next determined the expression levels of HIF proteins in CD70^+^/CD70^–^ cells to examine the involvement of the HIF pathways in the epigenetic regulation and induction of CD70 under hypoxia. Interestingly, HIF-2α levels were consistently higher in CD70^+^ populations of CaOV-2, PEO1 and H1975 cells compared to CD70^–^. Higher HIF-1α expression in CD70^+^ cells was only detected in PEO1 (Figure 4A). Combined with the CD70 promoter demethylation under hypoxia described above, this led us to hypothesize that HIF-2α may be a key transcription factor that mediates CD70 up-regulation via epigenetic derepression, which consequently leads to cancer cell aggressiveness. To address this, we established HIF-2α knockdown (KD) cells as well as HIF-1α-KD based on CD70^+^ PEO1 cells. The two independent HIF-2α-KD but not HIF-1α-KD reduced CD70^+^ expression under hypoxia (Figure 4B and Supplementary Figure 2C). Of note, one of the known epigenetic targets of HIFs is DNMT1, which is reported to down-regulate CD70 [44]. Thus we examined the effect of each two independent shRNA against HIF-1α or HIF-2α on *DNMT1* mRNA expression in hypoxia-treated cells. DNMT1 levels were decreased by 64–81% upon the 2-day hypoxia (1% O_2_) treatment in shNT cells or HIF-1α-KD while the changes were limited (16–19%) in HIF-2α-KD cells (Figure 4C). These data collectively suggest that HIF-2α plays an important role for CD70 up-regulation via DNMT1 suppression. Importantly, the colony forming efficiency of the two independent HIF-2α-KD cells in soft agar was significantly decreased compared to the control non-targeting (NT) KD (Figure 4D). In addition, high HIF-2α expression was also associated with poor prognosis in the ovarian cancer patients (Figure 4E). Taken together, these data demonstrated a regulatory link between HIF-2α function and CD70 expression, which promotes cancer cell proliferation. Our studies collectively suggest that HIF-2α elicits CD70 and this is associated with epigenetic derepression via DNA methylation. Therefore CD70 is also a marker of cancer aggressiveness, and growth advantage in diverse cancer types.

## DISCUSSION

We have identified a role for HIF-2α in the hypoxic regulation of the cancer marker CD70 that occurs through DNA methylation mediated by DNMT1 and drives cancer cell proliferation. We demonstrated the impact of CD70 expression on anchorage-independent growth in ovarian, lung, brain and kidney cancer cells. We showed that both hypoxia treatment and DNMT1 inhibition decreased the promotor methylation of CD70 that leads to its induction in pure CD70^–^ cells and that down-regulation of DNMT1 under hypoxia is mediated by HIF-2α but not HIF-1α. Importantly, both HIF-2α and CD70 expressions were correlated with poor prognosis of cancer patients.

CD70 expression has been increasingly linked to carcinomas or solid tumors while it is not expressed in intact non-lymphoid cells. Previous works have implicated CD70 function in growth advantage [45], drug resistance [28] and immune-escape [46]. In agreement with this, our data showed that CD70 is expressed in many ovarian, lung, brain and kidney carcinoma cells and CD70^+^ cells also have increased colony forming activity in soft agar compared to CD70^–^ cells (Table. 1). Anchorage-independent growth is a feature of cancer cells that are resistant to loss of anchorage which leads to cell cycle defect and anoikis (programmed cell death induced by loss of anchorage) [47]. These characteristics are important for metastasis and the epithelial-mesenchymal transition (EMT) [48] that constitute some of the properties of cancer stem-like cells [49]. Therefore, dysregulation of CD70 by HIF-2α, where either factor is able to enhance anchorage-independent growth (Figures 2A, 2B and 4D), may be a relevant feature of cancer stem/initiating cells. Although CD70 expressions in solid cancer stem cells have not been reported, our studies may suggest CD70 antigen as a potential cancer stem cell marker. This is supported by a recent report showing that CD70 signaling was involved in AML stem cell regulation [50].

Although both HIF-1α and HIF-2α have long been thought to promote tumor growth, their respective allocated roles have remained ambiguous due to their overlapping and complementary functions. With regard to physiological adaptation to low oxygen conditions, HIF-1α has been better studied compared to HIF-2α. In particular, accumulating evidence indicates the involvement of HIF-1α in epigenetic regulations [16, 17, 42]. In contrast, HIF-2α expression has been more heavily implicated in cancer progression [51, 52]. Interestingly, we found that silencing of either HIF-1α or HIF-2α resulted in decreased colony numbers in soft agar (Figure 4D, Supplementary Figure 2A) while others have shown that this led to attenuated spheroid growth [8]. We also showed that HIF-2α is primarily responsible for the regulation of CD70 expression. This implies that both HIF proteins are involved in anchorage-independent growth but this may occur via different pathways. A recent report showed that CD70 was up-regulated in VHL-mutated cells, in which HIF protein degradation is abolished, and this was mediated both by HIF-1α and HIF-2α [53]. The same study showed that the CD70 promoter was bound by HIFs and silencing of the HIFs led to slight changes in CD70 mRNA levels. The increases in CD70 protein levels in renal cell carcinoma cells they showed was likely more dependent on oxygen levels rather than the expression levels of HIFs, offering a possibility of the involvement of HIF-independent pathways. In the present study, on the other hand, HIF-2α is highly expressed in the sorted and hypoxia-treated CD70^+^ cells and the suppression of HIF-2α expressions led to the decrease of CD70 protein expressions in 5 days under low oxygen conditions (Figure 4A and 4B). HIF-1α is expressed ubiquitously and was induced by hypoxia in CaOV-2 and H1975 cells regardless of CD70 expression while the HIF-1α expression levels in PEO1 CD70^+^ cells were higher than that in CD70^–^ (Figure 4A). Importantly, the silencing of HIF-1α in the CD70^+^ cells did not significantly change CD70 expression while it was consistently diminished by HIF-2α-KD (Figure 4B). On the other hand, DNMT1 expression levels decreased under hypoxia were rescued not by HIF-1α-KD but by HIF-2α-KD. Taken together, HIF-1α is not likely responsible for the DNMT1-dependent epigenetic regulation of CD70 under hypoxia. Therefore, the regulation of CD70 may be a non-redundant function of HIF-2α. The consistency of hypoxia and azacitidine treatment in inducing the production of CD70^–^expressing cells from CD70^–^ cells suggests a potential involvement of epigenetic alteration in CD70 regulation (Figure 3B–3D). Of note, downstream changes in protein expression by epigenetic modifications may involve a time lag. Indeed, CD70 mRNA levels or protein expression were constant within 24 hours or 4 days of hypoxia treatment, respectively (Supplementary Figure 1D and 1E) while the changes in CD70 protein levels during HIF-2α knockdown were still subtle on day 2 (Supplementary Figure 2B). These observations are consistent with the notion that HIF-2α may not directly regulate CD70 expression via its transcriptional activity but indirectly through epigenetic modifications as DNA methylation/demethylation cycles may require a longer duration to alter gene expressions compared to transcription factors. In contrast to HIF-1α that has downstream target DNMTs and histone demethylases, the mechanisms that mediate epigenetic modifications via HIF-2α pathways remain unidentified. It is possible that HIF-2α also alters epigenetic status via other DNMTs which is supported by our preliminary data showing that DNMT3A protein levels were increased by hypoxia treatment in OVTOKO cells (Supplementary Figure 2D) and up-regulated in CD70^+^ H1975 cells compared to CD70^–^ cells (Supplementary Figure 2E). Recently, it has been suggested that DNMT1, DNMT3A and DNMT3B participate in the ‘rewriting’ of epigenetic marks [54]. Therefore, the up-regulation of DNMT3A expressions may indicate dynamic changes in DNA methylation status under hypoxia or in CD70^+^ cells. Of note, DNMT3A and 3B were reported to be involved in DNA demethylation [55]. Together, this suggests that active turnover of DNA methylation under conditions of hypoxia may elicit epigenetic reprogramming that leads to CD70 up-regulation. Conversely, while the involvement of DNMT3A in HIF-2α activation has been reported [56], it remains unclear whether it is HIF-1α, HIF-2α or both HIFs that regulate DNMT3A expression. In addition to DNA methylation, histone modifications are involved in the epigenetic regulation of the cancer surface markers CD44 or CD133 [57, 58]. While HIF-1α, rather than HIF-2α, has been implicated in chromatin remodeling, our studies suggest that HIF-2α can also be associated with histone modifications as methylated DNA recruits histone deacetylases [59]. Thus, the extent of HIF-2α functions in epigenetic regulation remains to be addressed in future studies.

In conclusion, we have identified a regulatory mechanism by which the oncogenic driver HIF-2α up-regulates CD70, a cell surface protein that is reflective of aggressive cancer cells. We showed that loss of either CD70 or HIF-2α significantly attenuated anchorage-independent growth (Figures 2B and 4D). Along with the patient data showing that high expression of these factors is correlated with poor patient prognosis (Figure 1A–1D, and Figure 4E), these results suggest that CD70 or HIF-2α can be promising targets for cancer therapeutics. In fact, molecular targeted therapy against both CD70 and HIF-2α is currently undergoing clinical trials with antibodies against CD70 for renal cell carcinoma, melanoma, lymphomas and other cancers as well as a HIF-2α inhibitor for renal cell carcinoma. Interestingly, many of these studies are conducted against renal cell carcinomas where constitutive HIF-2α activation is frequently observed due to VHL mutations [60] and CD70 is highly expressed [61]. This supports a probable epigenetic link between HIF-2α function and CD70 expression that we have identified in this study, providing a potential avenue for coordinated drug treatment against both factors.

## MATERIALS AND METHODS

### Cell culture

The ovarian cancer cells OVTOKO (JCRB Cell Bank, Japan, Ibaraki, Japan), OVISE (JCRB Cell Bank), PA-1 (ATCC, Manassas, VA, USA), PEO1 (CRT, Coralville, IA), TOV-112D (ATCC), TOV-21G (ATCC), lung cancer A549 (ATCC), H1299 (ATCC), H1975 (ATCC), HCC2935 (ATCC), PC-14 (ECACC, Salisbury, UK) and glioma LN-229 (ATCC) cell lines were grown in RPMI-1640 media supplemented with 2 mM L-glutamine (Nacalai Tesque, Suita, Japan) and 10% FBS (Serana, Pessin, Germany, or Sigma-Aldrich, St. Louis, MO, USA). CaOV-2 and HEYA8 cells were obtained from SGOCL(43), a previously introduced ovarian cancer cell line library [62], and also cultured in RPMI-1640 media described above. The ovarian cancer cell SKOV3 (ATCC), glioma A172 (ATCC), renal cell carcinoma cells 786-O (ATCC), and A498 (ATCC) were grown in DMEM media (Nacalai Tesque) containing 10% FBS and 2 mM Glutamax (Thermo Fisher, Waltham, MA). All cell lines used in this study were authenticated by the Centre for Translational Research and Diagnostics (CTRAD, Singapore) or certified by ATCC within 5 years. For routine mycoplasma testing, the MycoAlert Mycoplasma Detection Kit (Lonza, Basel, Switzerland) was employed in the course of this study. Cells were incubated in a Forma Steri-Cycle CO_2_ incubator (Thermo Fisher) at 37° C with 5% CO_2_ and ambient oxygen level (approximately 20.9% O_2_). Hypoxia treatments were performed in an *Invivo*2 hypoxia workstation (Baker Ruskinn, Sanford, ME, USA) at 37° C in a 5% CO_2_, 1% O_2_ and N_2_-balanced atmosphere. For DNMT1 inhibition, 5-azacitidine (Sigma) or DMSO vehicle control (Sigma) were added to the media for the durations as indicated.

### Soft agar assays

Trypsinized cells were resuspended in RPMI-1640 media without phenol red (#R8755, Sigma) containing 0.35% low-melt agarose (#1613111, Bio-Rad, Hercules, CA, USA) and 10% FBS (Sigma). 2,000 cells suspended in agarose-containing media were plated on top of a 0.5% agarose base layer. The cells were cultured in a Forma Steri-Cycle CO_2_ incubator (Thermo Fisher) CO_2_ incubator at 37° C with 5% CO_2_ for 3 weeks and stained with 0.05% crystal violet (Gentian Violet, ICM Pharma, Singapore). For image capture of colonies, a dissection microscope (SZX-12, Olympus, Tokyo, Japan) was used while the colony numbers and sizes were quantitated using the Image J software [63].

### Flow cytometry

Cells were trypsinized and resuspended in Hank’s balanced salt solution (HBSS) containing 2% BSA followed by staining with anti-CD70 PE conjugate antibody (555835, BD BioSciences, San Jose, CA, USA) and SYTOX-Blue (Thermo Fisher) for dead cell staining. Cells were sorted using the BD FACSAria or analyzed using the BD LSR II with the BD FACSDiva Software (BD BioSciences).

### RNAi

The vectors used for stable mRNA knockdowns based on pLKO.1 backbone with shRNAs against HIF1A (HIF-1α #1; TRCN0000003811 and HIF-1α #2; TRCN0000010819) and EPAS1 (HIF-2α #1; TRCN0000003806 and HIF-2α #2; TRCN0000003807) were purchased from Sigma-Aldrich (see Supplementary Table 1). For lentivirus productions, 293T cells (ATCC) were co-transfected with the shRNA and with packaging vectors and incubated overnight. The transfection media was replaced with complete growth media (DMEM containing 10% FBS) and the resulting supernatant was collected 48 hours later followed by filtration with 0.45 µm-pore syringe filters (Sartorius, Göttingen, Germany) and stored at −80° C. For infection, typically, 400,000 cells were infected with 4 mL of the virus supernatant with polybrene (8 µg/mL, Sigma) for 4 hours. Puromycin (Sigma) was added at 2 ng/mL 48 hours after infection for the establishment of stable cell lines. For CD70 knockdown, Silencer^®^ Select siRNA-CD70 (Thermo Fisher) against *CD70* was transfected using Lipofectamine^®^ RNAiMAX reagent (Thermo Fisher) according to the manufacturer’s instructions. Briefly, 10 pmol of the reconstituted siRNA oligo was mixed with 3 µL of the Lipofectamine^®^ RNAiMAX and added onto CD70^+^ PEO1 cells with 100 µL of Opti-MEM^®^ I reduced serum media in 12-well plates (Greiner bio-one, Kremsmünster, Austria) followed by 4 hours of incubation at 37° C with 5% CO_2_. The transfection media was changed to RPMI-1640 media and the cells were allowed to grow for a further 24 hours after transfection before being used for subsequent experiments.

### Immunoblotting

Cells were harvested and lysed in M-PER reagent (Thermo Fisher) containing cOmplete^™^ protease inhibitor cocktail (Roche, Basel, Switzerland) and PMSF (Sigma). Each 10 to 15 µg of denatured protein samples were separated on Mini-PROTEAN^®^ TGX^™^ Precast Gels (Bio-Rad) and transferred to methanol-activated Hybond P membranes (GE Healthcare, Pittsburgh, PA, USA). Membranes were blocked with TBS-T buffer containing 5% skimmed milk and incubated with anti-α-tubulin (sc-23948, Santa Cruz, Dallas, TX, USA), anti-DNMT3A (3598, Cell Signaling, Danvers, MA, USA), anti-HIF-1α (GTX127309, GeneTex, Irvine, CA, USA) or anti-HIF-2α (7096, Cell Signaling) antibodies. For detection of protein expression with multiple antibodies, membranes were cut into up to 3 pieces with a sufficient margins. Bands from multiple blots in a single experiment were normalized by Ponceau-S staining (Sigma-Aldrich) Supplementary Figure 3A–3F.

### Quantitative RT–PCR

Total RNA was extracted using the RNeasy mini kit (Qiagen, Hilden, Germany) and cDNA was synthesized from 1 µg of total RNA with random hexamers using the RevertAid First Strand cDNA Synthesis Kit (Thermo Fisher) according to the manufacturer’s instructions. Quantitative RT–PCR was performed using the KAPA SYBRFast qPCR kit (Kapa Biosystems, Wilmington, MA, USA) on the 7500 Fast Real-Time PCR System (Applied Biosystems, Foster City, CA, USA). PCR reactions were carried out in biological triplicates and technical triplicates (*n* = 9) and relative expressions were calculated using the comparative CT method with B2M expression as the reference control. (See Supplementary Table 1 for primer sequences.)

### Bisulfite sequencing

Genomic DNA was extracted with ReliaPrep^™^ gDNA Tissue Miniprep System (Promega, Fitchburg, Wisconsin) from cell line samples in triplicate, and treated with EpiTect Bisulfite Kit (Qiagen). For bisulfite sequencing, PCR reaction was performed to amplify the proximal promoter region of CD70 gene with the primer sets listed in Supplementary Table 1. PCR products were sub-cloned with DynaExpress TA PCR cloning kit (BioDynamics Laboratory Inc., Tokyo, Japan) and sequenced with a BigDye terminator v3.1 cycle sequencing ready reaction kit (Applied Biosystems Japan Ltd., Tokyo, Japan) and an ABI PRISM 3100 genetic analyzer (Applied Biosystems Japan). For each sample, ten clones were sequenced.

### Clinical data analyses

KM plotter (kmplot.com) [37] for ovarian, lung, breast and gastric cancer and REMBRANDT (betastasis.com) [64] for glioma were used for determination of Kaplan-Meier survival curves.

### Statistical analyses

Independent one-sample and two-sample *t*-tests were performed as indicated.

## SUPPLEMENTARY MATERIALS FIGURES AND TABLE


