# Supplementary material for: Hypoxia-inducible factor-2 alpha up-regulates CD70 under hypoxia and enhances anchorage-independent growth and aggressiveness in cancer cells
Source: Oncotarget. 2018 Apr 10;9(27):19123–35. doi: 10.18632/oncotarget.24919 (PMC5922382; doi:10.18632/oncotarget.24919)
Supplement: Supplementary file 1 [file oncotarget-09-19123-s001.pdf]

Hypoxia-inducible factor-2 alpha up-regulates CD70 under hypoxia and enhances anchorage-independent growth and aggressiveness in cancer cells

SUPPLEMENTARY MATERIALS

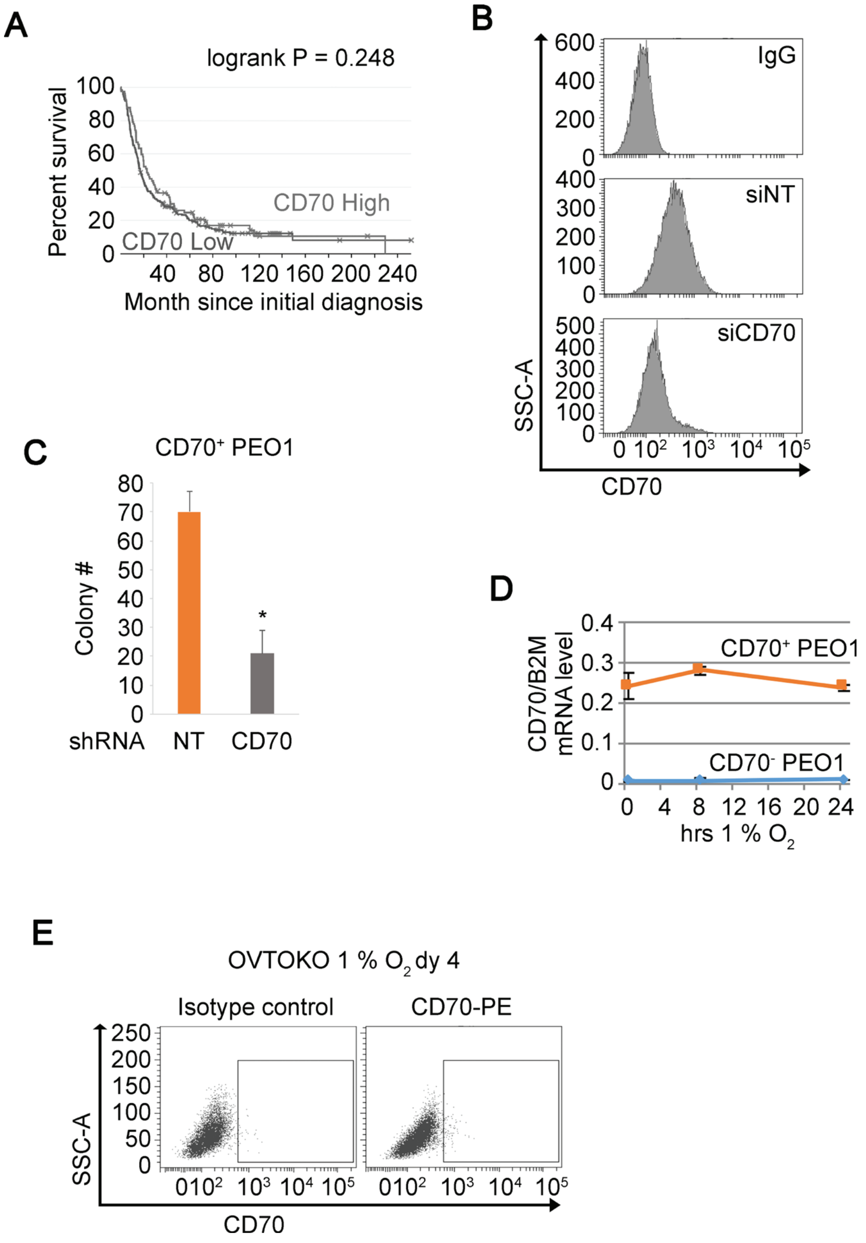

Supplementary Figure 1: (**A**) Kaplan–Meier plot of high and low CD70-expressing glioma patients. Overall survival is shown. (**B**) The CD70 expressions in CD70$^+$ PEO1 transfected with siRNA-CD70 (siCD70) or non-targeting control siRNA (siNT) were analyzed by flow cytometry (**C**) Changes in colony number in soft agar by a stable CD70 knockdown using shRNA. NT, non-targeting control. (**D**) *CD70* mRNA levels normalized to B2M housekeeping reference gene under hypoxia were analyzed by qRT-PCR in CD70$^+$/CD70$^-$ PEO1 cells in the indicated time course. (**E**) OVTOKO cells were cultured under 1% O_2 conditions for 4 days and stained with CD70 antibody or isotype control. Plots show CD70 expressions detected by flow cytometry. Error bars indicate s.e.m. $^*P < 0.05$ (Student's *t*-test).

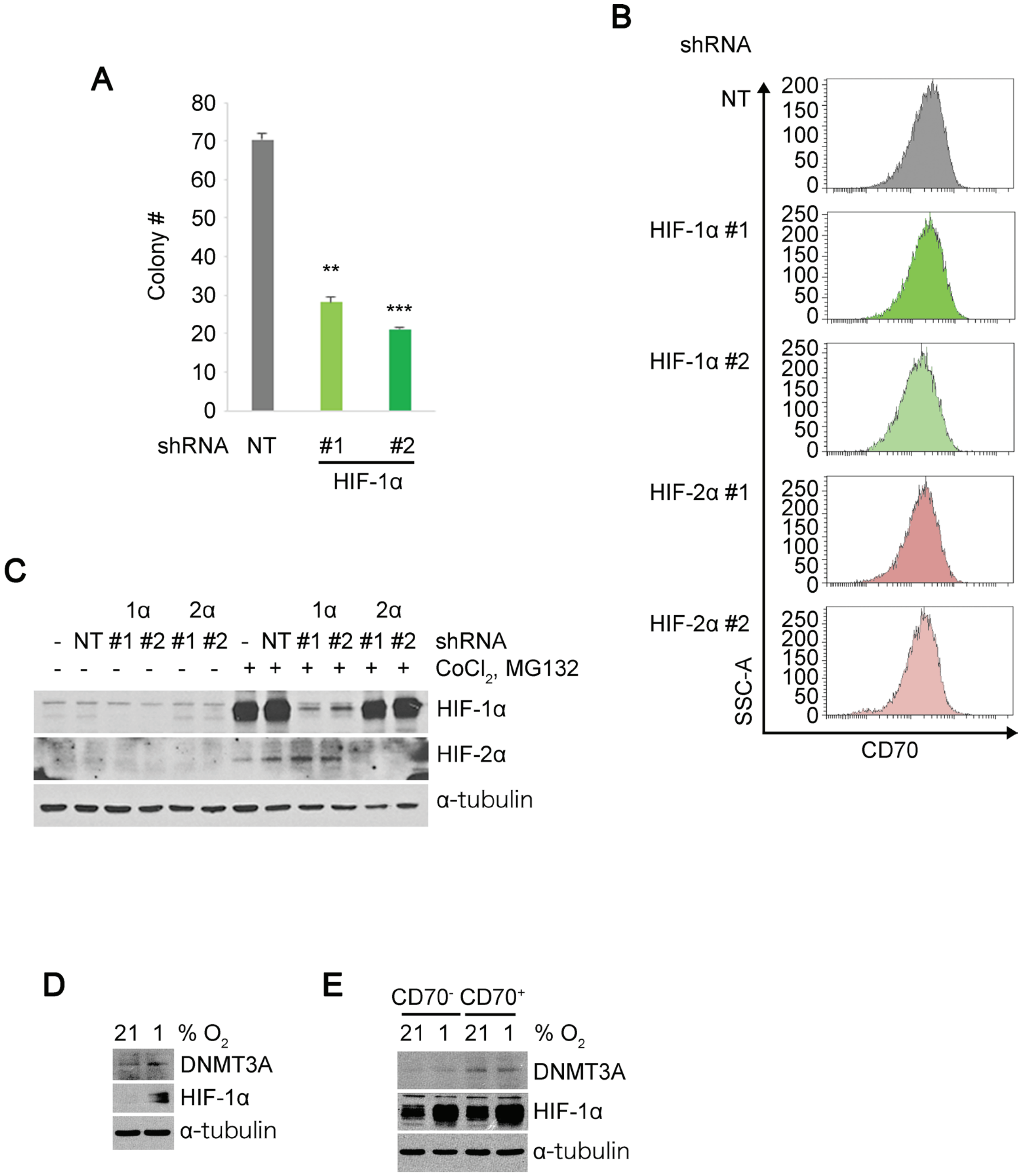

Supplementary Figure 2: (**A**) Colony numbers in soft agar of two independent HIF-1α knockdown together with non-targeting (NT) control. (**B**) CD70 protein expressions in CD70+ PEO1 cells with NT control, HIF-1α or HIF-2α shRNA knockdown cultured under hypoxic conditions for 2 days were analyzed by using flow cytometry. (**C**) Immunoblots showing HIF-1α and HIF-2α levels in CD70+ PEO1 cells infected with each two independent HIF-1α (1α) or HIF-2α (2α) shRNAs compared to NT control. Cells with the indicated shRNAs were treated in the presence (+) or absence (−) of $CoCl_2$ and MG132. α-tubulin expressions was used as a protein loading control. (**D**, **E**) DNMT3A expression under hypoxia in OVTOKO (D) or H1975 (E) cells was examined by immunoblot. HIF-1α or α-tubulin (those in panel E and Figure 4A are identical) were used for hypoxia or loading controls, respectively.

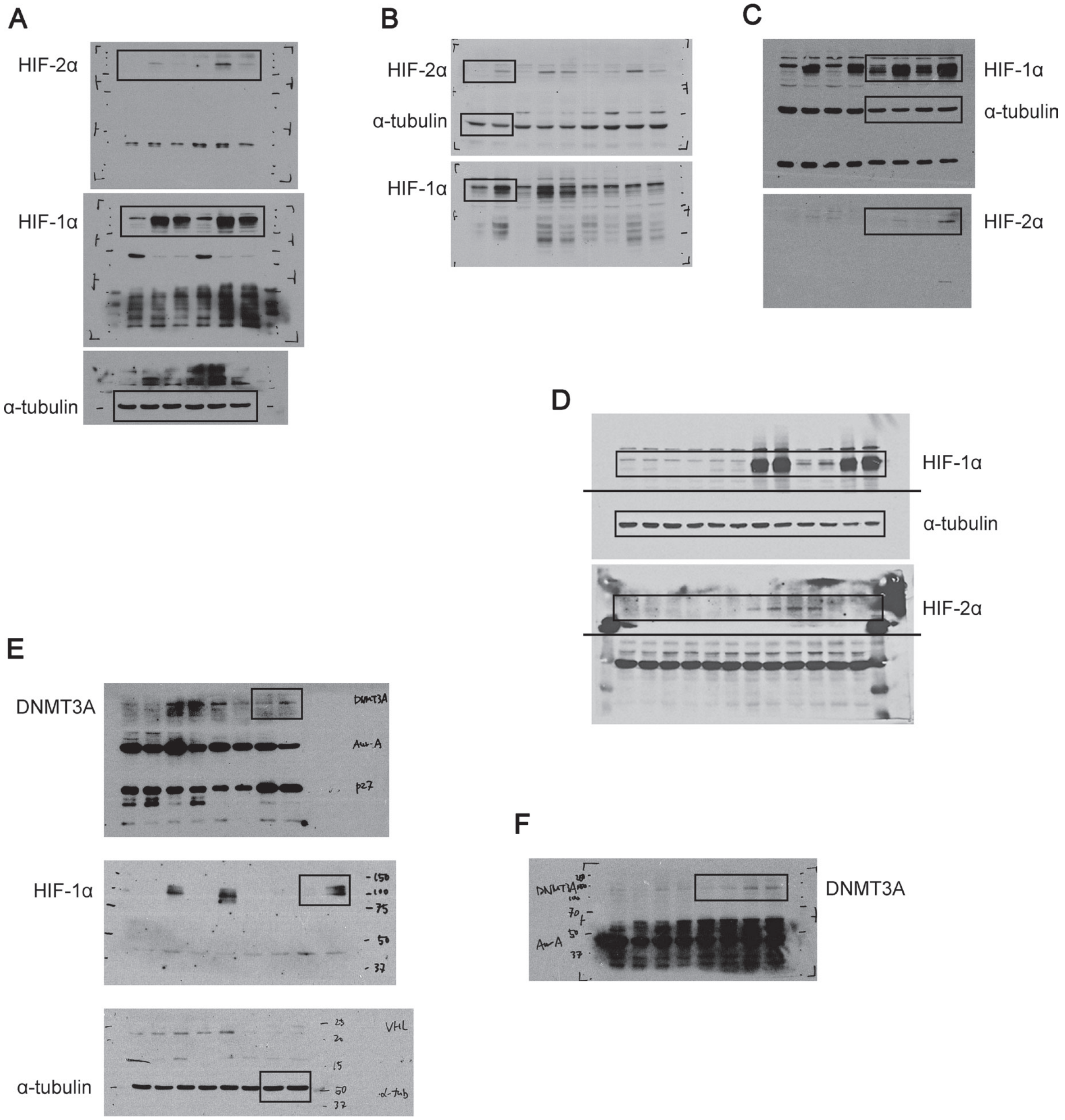

Supplementary Figure 3: Uncropped images of immunoblots. (**A–C**) HIF-2α, HIF-1α and α-tubulin protein expression levels are indicated in CaOV-2 (A), PEO1 (B) or H1975 (C) cells as represented in Figure 4A. (**D**) HIF-1α, α-tubulin and HIF-2α expressions for Supplementary Figure 2C. (**E**) DNMT3A, HIF-1α and α-tubulin immunoblots are shown as for Supplementary Figure 2D. (**F**) DNMT3A protein expression as indicated in Supplementary Figure 2E. The blots showing HIF-1α and α-tubulin expression levels in H1975 for Supplementary Figure 2E were identical to those shown in Figure 4A.

Supplementary Table 1: The target sequences for qRT-PCR, bisulfite sequencing and RNAi

Primer sequences

Target	Symbol		Sequence
CD70	*CD70*	Foward	TCTCAGCTTCCACCAAGGTT
CD70	*CD70*	Reverse	AAGTGTCCCAGTGAGGTTGG
B2M	*B2M*	Foward	TGCTCGCGCTACTCTCTCTTT
B2M	*B2M*	Reverse	TGTCGGATGGATGAAACCCAGA
CD70*	*CD70*	Foward	TTAAAGAGGAAGTAGGTTTGAATTAG
CD70*	*CD70*	Reverse	TCAAAAACTACTAAAAACTTCACAAAACTAAA

*for bisulfite sequencing.

shRNA target sequences

Target	Symbol		Sequence	Region
CD70	*CD70*	#1	CCGGGAAACACTGATGAGACCTTCTCTCGAGAGAAGGTCTCATCAGTGTTTCTTTTT	CDS
HIF-1α	*HIF1A*	#1	CCGGCGGCGAAGTAAAGAATCTGAACTCGAGTTCAGATTCTTTACTTCGCCGTTTTT	CDS
HIF-1α	*HIF1A*	#2	CCGGTGCTCTTTGTGGTTGGATCTACTCGAGTAGATCCAACCACAAAGAGCATTTTT	3′ UTR
HIF-2α	*EPAS1*	#1	CCGGCAGTACCCAGACGGATTTCAACTCGAGTTGAAATCCGTCTGGGTACTGTTTTT	CDS
HIF-2α	*EPAS1*	#2	CCGGCCATGAGGAGATTCGTGAGAACTCGAGTTCTCACGAATCTCCTCATGGTTTTT	CDS
